# Annotation data about multi criteria assessment methods used in the agri-food research: The french national institute for agricultural research (INRA) experience

**DOI:** 10.1016/j.dib.2019.104204

**Published:** 2019-07-22

**Authors:** Geneviève Gésan-Guiziou, Aude Alaphilippe, Mathieu Andro, Joël Aubin, Christian Bockstaller, Raphaëlle Botreau, Patrice Buche, Catherine Collet, Nicole Darmon, Monique Delabuis, Agnès Girard, Régis Grateau, Kamal Kansou, Vincent Martinet, Jeanne-Marie Membré, Régis Sabbadin, Louis-Georges Soler, Marie Thiollet-Scholtus, Alberto Tonda, Hayo Van-Der-Werf

**Affiliations:** a STLO, INRA, Agrocampus Ouest, 35000, Rennes, France; b UERI-Gotheron, INRA, 26320, Saint-Marcel-lès-Valence, France; c DIST, INRA, 78000, Versailles, France; d SAS, INRA, Agrocampus Ouest, 35000, Rennes, France; e LAE, INRA, University of Lorraine, 68000, Colmar, France; f UMRH, INRA, University of Clermont, University of Lyon, VetAgro Sup, 63122, St Genes Champanelle, France; g IATE, University of Montpellier, INRA, 34060, Montpellier, France; h Silva, University of Lorraine, AgroParisTech, INRA, 54000, Nancy, France; i MOISA, INRA, CIRAD, CIHEAM-IAMM, SupAgro, Montpellier University, 34060, Montpellier, France; j LPGP, INRA, 35000, Rennes, France; k Economie Publique, AgroParisTech, INRA, University Paris-Saclay, 78850, Thiverval-Grignon, France; l BIA, INRA, 44300, Nantes, France; m SECALIM, INRA, Oniris, University Bretagne Loire, 44300, Nantes, France; n MIA, INRA, 31320, Castanet Tolosan, France; o INRA, 75007, Paris, France; p ASTER, INRA, 68000, Colmar, France; q GMPA, INRA, AgroParisTech, University Paris-Saclay, 78850, Thiverval-Grignon, France

**Keywords:** Literature search query, INRA divisions, Trade-offs, Multicriteria decision, Multicriteria assessment

## Abstract

This data article contains annotation data characterizing Multi Criteria Assessment (MCA) Methods proposed in the agri-food sector by researchers from INRA, Europe's largest agricultural research institute (INRA, http://institut.inra.fr/en). MCA can be used to assess and compare agricultural and food systems, and support multi-actor decision making and design of innovative systems for crop production, animal production and processing of agricultural products. These data are stored in a public repository managed by INRA (https://data.inra.fr/; https://doi.org/10.15454/WB51LL).

**Table:** Specifications Table

Subject area	Multi Criteria Assessment (MCA) Methods
More specific subject area	*Agri-food sector*
Type of data	*Table*
How data was acquired	*A collection of 954 scientific papers from 2007 to 2017 extracted from Web Of Science (WOS) using two keywords WOS search queries, published by INRA researchers belonging to 13 scientific domains listed in*Table 1*and annotated using 8 major characteristics.*
Data format	*Raw and analyzed.*
Experimental factors	*Classification of scientific papers in MCA or non-MCA is defined in this article*
Experimental features	*Classification of scientific papers in 8 characteristics (Type of study, Purposes, Audience, Assessed dimensions, Assessed system/object; Spatial scale, Time scale, Actors' contribution) is defined in this article.*
Data source location	*INRA, FR-75000, Paris, France*
Data accessibility	*Data are accessible in a public repository* (https://data.inra.fr/; https://doi.org/10.15454/WB51LL)

**Table:** 

**Value of the data** • A unique set of annotation data about Multi Criteria Assessment Methods proposed in the scientific literature in the agri-food sector • These data can be used to analyze Multi Criteria Assessment Methods in a large spectrum of activities in the agri-food sector • These data could serve as benchmark for researchers coping with Multi Criteria Assessment Methods in the agri-food sector or other fields of activity

## 1 Data

Scientific articles dealing with Multi Criteria Assessment (MCA) have been associated with annotations. These scientific papers have been extracted from the WOS using two WOS key-words queries (see section 2.1), and manually typed MCA or non MCA by a group of INRA experts (see section 2.2). The MCA articles have finally been classified according to 8 major characteristics (Type of study, Purposes, Audience, Assessed dimensions, Assessed system/object; Spatial scale, Time scale, Actors’ contribution), each of them being divided into several categories (see section 2.3).

These data (954 papers) have been grouped in 1 global Excel file, and split into 13 Excel files corresponding to scientific domains to cover the diversity of the disciplinary approaches and applications developed in INRA (Table 1). Redundancies may exist between the 13 Excel files as some articles may appear in several scientific domains.

**Table 1:** List of articles grouped by application domain.

Domain	Table DOI	Amount of MCA articles	Amount of non-MCA articles
Agri-food (global file)	https://doi.org/10.15454/WB51LL	954	4920
Food and Bioproduct Engineering	https://doi.org/10.15454/R2E0XD	265	560
Nutrition, Chemical Food Safety and Consumer Behaviour	https://doi.org/10.15454/XQONFE	112	617
Environment and agronomy	https://doi.org/10.15454/RSFBTX	248	1404
Animal Physiology and Livestock Systems	https://doi.org/10.15454/YOZ5LV	236	940
Animal Genetics	https://doi.org/10.15454/DACDJM	198	541
Social Science, Agriculture and Food, Rural Development and Environment	https://doi.org/10.15454/RZKKWG	169	497
Microbiology and the Food Chain	https://doi.org/10.15454/FUGVMT	160	518
Science for Action and Development	https://doi.org/10.15454/TW5WAX	115	436
Plant Health and Environment	https://doi.org/10.15454/3SI1GB	101	967
Plant Biology and Breeding	https://doi.org/10.15454/T5J8EP	78	655
Animal Health	https://doi.org/10.15454/UTACZ6	49	331
Forest, Grassland and Freshwater Ecology	https://doi.org/10.15454/KTV4NG	45	870
Applied Mathematics and Informatics	https://doi.org/10.15454/VHDQB8	26	310

These data are stored in a INRA institutional data repository powered by Dataverse (https://data.inra.fr/).

## 2 Experimental design, materials, and methods

The annotation of scientific papers has been done in 3 steps. Firstly, the set of papers have been extracted from the WOS using a set of key-words. These search queries were performed mid 2017. The resulting corpus of papers (4920 papers) has been manually typed MCA (954 papers) or non MCA (3966 papers) by domain experts using a set of positive and negative criteria defining the notion of MCA articles. In the last step, MCA-typed articles have been annotated using 8 characteristics and associated modalities.

### 2.1 Step 1 WOS search queries

Two queries have been created to extract articles from the WOS. The first one target articles which refers explicitly to MCA methods. The second one aims papers which propose methods to compare alternatives or directly compare alternatives using several criteria without explicit references to MCA methods.

Query 1 (MCA explicit): Field1 AND Field2 AND Field3 with:

Field1/* Address INRA */

(AD=((France or guad* OR fr* guian* OR kourou OR french* OR Fr pol* OR belg* OR W Ind Assoc St) SAME ((FRENCH INST AGR & FOOD RES* CTR) OR (FRENCH INST AGR* RES*) OR (FRENCH NAT* INST AGR SCI) OR (INCRA) OR (INR4) OR (INRA) OR (INST NACL RECH AGR*) OR (INST NAT* AGR* RES) OR (INST NATL DE LA RECH AGRON) OR (INST NATL RECH A GRONOM) OR (INST NATL RECH AGNON) OR (INST NATL RECH ARGONOM) OR (INST NATL RECH ARON) OR (INST NATL SUPER RECH AGRON) OR (INST* RECH* AGRON*) OR (INST REC* NAT* AGR*) OR (INST SCI RECH AGR*) OR (INST* NAT* REC* AGR*) OR (INST* NAT* RES* AGR*) OR (INT INST AGR* RES*) OR (LINST NAT* REC* AGR*) OR (NAT* INST* AGR* RES*) OR (NAT* AGR* RES) OR (NAT* INST RES* AGR*) OR (NAT* RE* INST AGR*) OR (NRA))))

**Fig. 1:**
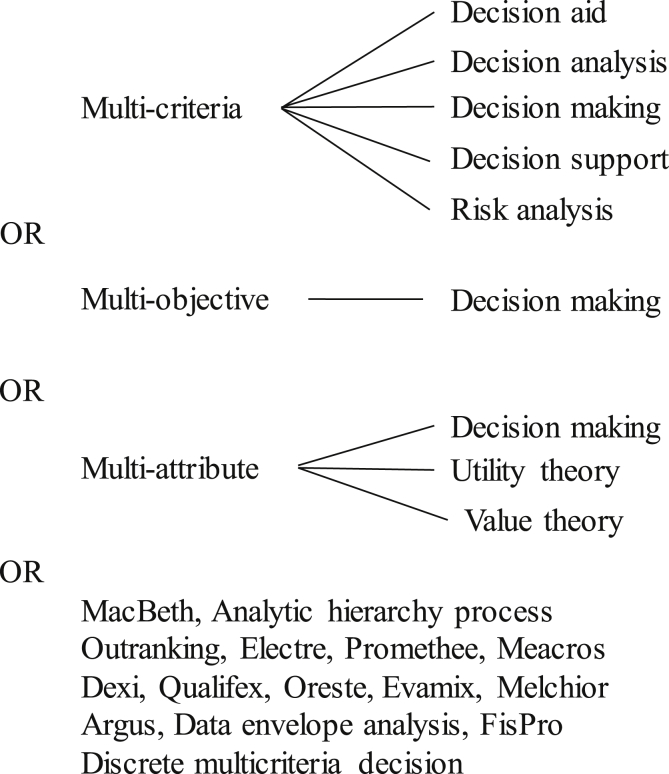
Diagram describing the distribution of keywords for the Field 2 (Topic) of Query 1.

Field2/* Topic */(see also Fig. 1).

MCDA OR "multi-criter* decision-aid*" OR "multicriter* decision-aid*" OR "multiple criter* decision-aid*" OR "multi-criter* decision-analy*" OR "multicriter* decision-analy*" OR "multiple-criter* decision-analy*“ OR MCDM OR "multi-criter* decision-making" OR "multicriter* decision-making" OR "multiple criter* decision-making" OR MODM OR "multi-objective* decision-making" OR "multiple objective* decision-making" OR MADM OR "multi-attribute* decision-making" OR "multiple attribute* decision-making" OR MCDS OR "multi-criter* decision-support" OR "multicriter* decision-support" OR "multiple criter* decision-support" OR MCRA OR "multi-criter* risk-analy*" OR "multicriter* risk-analy*" OR "multiple criter* risk-analy*"OR MAUT OR "multi-attribute* utility-theor*" OR “multiple attribute utility theor*" OR MAVT OR "multi-attribute* value-theor*" OR "multiple attribute* value-theor*" OR MACBETH OR AHP OR "analytic* hierarch* process*" OR Outranking OR ELECTRE* OR PROMETHEE OR MEACROS OR DEXi* OR DEX OR QUALIFLEX OR ORESTE OR EVAMIX OR MELCHIOR OR ARGUS OR DEA OR "data-envelop* analy*" OR FisPro OR DMD OR "Discrete multicriter* decision*" OR "Discrete multi-criter* decision*“

Field 3/* Period */

year: 2007–2017.

Query 2 (non MCA explicit): Field1 AND Field2 AND Field3 AND Field4 with:

Field1/* Address INRA, same as in Query 1 */

Field2/* Topic */evaluat* OR Assess* OR decision OR optim* OR design OR selection*

Field3/* Topic */

(indicator* OR "multi-criter*" OR multicriter* OR criter* OR "risk-benefit" OR riskbenefit OR ranking OR "multi-agent*" OR multiagent* OR scenari* OR option* OR "reference value*" OR LCA OR "life cycle analy*" OR "lifecycle analy*" OR "lifecycle assess*" OR "life-cycle assess*" OR LCAs OR performanc* OR "cost-benefit" OR costbenefit OR "trade-off*" OR "trade off*" OR tradeoff* OR aggregat* OR "multi-attribut*" OR multiattribut* OR "multi-perform*" OR multiperform* OR "multi-objectiv*" OR multiobjectiv* OR "multi-funct*" OR "multifunct*" OR "multi-goal*" OR multigoal* OR "linear-program*" OR argumentation OR arbitration* OR viewpoint* OR "view-point*" OR "fuzzy logic" OR "decision-tree*" OR viab* OR "operational research" OR preferenc* OR Pareto OR "environmental impact assess*" OR sustainab* OR "decision support system*" OR "decision-analys*" OR "utility-theor*" OR "scoring")

Field 4/* Period */

**year**: 2007–2017.

### 2.2 Step 2 selection of MCA articles

A set of positive and negative criteria has been defined and used to classify articles extracted from the WOS in step 1 as MCA or Non-MCA.

The papers have been classified as Non-MCA if they present a study of one of the following types:

1. A descriptive study based on a set of variables/indicators not interpreted in terms of comparison of alternative scenarii;
2. The design of a phenomena predictive model (statistical, numerical, …) except if it is explicitly integrated in a broader approach of multicriteria assessment;
3. A mono-objective optimisation study without constraints.

The papers have been classified as MCA if they present a study of one of the following types:

4. A study of several alternatives based on several criteria/indicators with interpretation (hierarchisation, ranking, comparisons,), even criterion by criterion;
5. A study based on the design of aggregated indicators representing a phenomenon/concept non measurable;
6. A multi-objective optimisation study or mono-objective with constraints expressed on criteria;
7. A study about methodologies/methods explicitly linked to a MCA approach;
8. Strategic/opinion paper about MCA approaches or issues requiring MCA methods;
9. A study which identifies a list of criteria taken into account to assess a system/property/concept.

Classification has been done in two steps. In each scientific domain (see Table 1), a double-blind annotation has been done on at least 50 randomly drawn articles to train the annotators/experts. During this first annotation step (consensus ranging from 70 to 92% between the annotators of a given scientific domain), the classification rules (MCA or non-MCA criteria) were defined; 2) The remaining set of articles has been annotated by at least one annotator.

### 2.3 Step 3 MCA articles characterization

The MCA articles have been classified according to 8 major characteristics, each of them being divided into several categories. In each Excel file, in the thumbnail "EMC_YES", columns A to P provide information about articles results of the bibliographic search. They have been filled automatically. Columns Q to BZ correspond to MCA articles characterization presented below.

1. **Type of study** (exclusive: one response only)

5 categories have been defined:

i) Specific methodological issues, which are not covered by choice ii) e.g., scale change, functional units, uncertainty management; Examples are study on sensitivity of DEXi-based decision tree [1]), spatial issue in Life Cycle Assessment [2];
ii) Development of generic methods: a method that does not propose a given list of criteria but makes it possible to define, choose, organize, aggregate or treat criteria. Examples are ELECTRE [3]; PROMETHEE [4] or development of decision support system [5];
iii) Development of methods dedicated to MCA: a method with its given criteria/indicators, its framework to organize and aggregate them. An example is the MASC method [6], developed from DEXi [7].
iv) Use/Application of method dedicated to MCA: articles applying method, without development, to case studies (articles using Life Cycle Assessment are in this type, see for instance [8]);
v) Applications with no dedicated method: applications using a list of indicators to compare options without a MCA method (this type includes, among other possibilities, studies examining different scenarii with respect to different criteria/indicators with some interpretation (hierarchy, typology, comparisons, etc.), even criterion by criterion, see for instance [9].
vi) Others: comparison of methods, reviews, position papers, see for instance [10], [11].

2. **Purposes** (definition of the objectives of the study) – Multiple choices possible

We used the classification of purposes adapted from Lairez et al. [12], [13].

7 categories:

i) To sensitize/structure actions (e.g., prioritize research actions)
ii) To deliver new knowledge (state evolution, comparison of systems), e.g., dashboards
iii) To report, e.g., on the achievement of a goal within an action plan (“external or internal reporting"), or on the compliance with regulation
iv) To identify elements of an option to improve: assessing the strengths and weaknesses of options
v) To choose, sort out, rank options [12], [13].
vi) To access to new market (e.g., getting a label)
vii) To promote (e.g., nutritional or environmental facts)

The two last categories are targeted when the MCA is oriented towards communication, with or without immediate benefits.

3. **Audience** – Multiple choices possible

The targeted audience is the one mentioned (or suggested) in the abstract of the paper and split in 6 categories

i) Scientists
ii) Development engineers (technical institutes, engineering consultants, chamber of agriculture, etc.)
iii) Farmers
iv) Industrials, processors, manufacturers
v) private and associate stakeholders (NGO, associations of consumers, associations of farmers, etc.)
vi) public stakeholders (Ministries, local government, EU, water agencies, environment agencies, health and safety agency, etc.)

4. **Assessed dimensions** – Multiple choices possible

8 categories

i) Functional and technical performances
ii) Economic
iii) Environmental
iv) Social
v) Product quality
vi) Human health (ex diet)
vii) Animal and plant health and welfare
viii) Ecosystem services

5. **Assessed system/object** – Multiple choices possible

10 categories

i) Plant
ii) Animal
iii) Plant and animal
iv) Micro-organisms
v) Processing
vi) Food
vii) Human organization: socio-economic institutions, farms, industrial sector…
viii) Health (pharmaceutical, medical, human genetics, etc.)
ix) Natural areas: ecosystem studied as a whole (fauna, flora, biodiversity, etc.). Examples: rivers, soils, forests, ….
x) Other, to be specified

6. **Spatial scale** – Multiple choices possible

6 categories

i) Individual (plant/animal/tree/unit operation/a specific food/bacteria, etc.)
ii) Collection of individuals, population (field/herd/forest stands/process/collection of food, etc.)
iii) System (farm/diet/forest/factory/ecosystem)
iv) Territory/supply chain
v) Nation/World regions (ex EU)
vi) Global

7. **Time scale** – Multiple choices possible (for example, LCAs can be both static and be applied to le life-cycle of production)

4 categories

i) Static: instantaneous picture of a system, or a temporal approach (could include comparative or repeated static analysis)
ii) Dynamic
iii) Scale of a production cycle (lifetime of an organism/human; production cycle...)
iv) Scale of the year
v) Several years; long-term

8. Contribution of **actors** – Multiple choices possible

An actor is defined as a person consulted for the study (other than the authors), including scientists consulted as experts, to define weights.

7 categories

i) Initial choice of the methods
ii) Definition of criteria and indicators
iii) interpretation of indicators (judgment, preferences, etc.)
iv) Opinion on aggregation (weighting, etc.)
v) Not specified
vi) Other, to be specified
vii) Irrelevant (no actor contribution)
